# Mutational analysis of genes coding for cell surface proteins in colorectal cancer cell lines reveal novel altered pathways, druggable mutations and mutated epitopes for targeted therapy

**DOI:** 10.18632/oncotarget.2374

**Published:** 2014-08-25

**Authors:** Elisa Donnard, Paula F. Asprino, Bruna R. Correa, Fabiana Bettoni, Fernanda C. Koyama, Fabio C.P. Navarro, Rodrigo O. Perez, John Mariadason, Oliver M. Sieber, Robert L. Strausberg, Andrew J.G. Simpson, Denis L.F. Jardim, Luiz Fernando L. Reis, Raphael B. Parmigiani, Pedro A.F. Galante, Anamaria A. Camargo

**Affiliations:** ^1^ Centro de Oncologia Molecular, Hospital Sírio-Libanês, São Paulo, Brazil.; ^2^ Programa de Pós Graduação do Departamento de Bioquímica, Instituto de Química, Universidade de São Paulo, São Paulo, Brazil.; ^3^ Laboratory of Molecular Biology and Genomics, Ludwig Institute for Cancer Research, São Paulo, Brazil.; ^4^ Instituto Angelita & Joaquim Gama, São Paulo, Brazil.; ^5^ Oncogenic Transcription Laboratory, Ludwig Institute for Cancer Research, Melbourne, Australia.; ^6^ Colorectal Cancer Genetics Laboratory, Systems Biology and Personalised Medicine Division, Walter and Eliza Hall Institute of Medical Research, Parkville, Australia.; ^7^ Faculty of Medicine, Dentistry and Health Sciences, Department of Medical Biology, University of Melbourne, Parkville, Australia; ^8^ Ludwig Institute for Cancer Research, New York, USA.

**Keywords:** colorectal cancer, targeted therapy, cell surface proteins, somatic mutations

## Abstract

We carried out a mutational analysis of 3,594 genes coding for cell surface proteins (Surfaceome) in 23 colorectal cancer cell lines, searching for new altered pathways, druggable mutations and mutated epitopes for targeted therapy in colorectal cancer. A total of 3,944 somatic non-synonymous substitutions and 595 InDels, occurring in 2,061 (57%) Surfaceome genes were catalogued. We identified 48 genes not previously described as mutated in colorectal tumors in the TCGA database, including genes that are mutated and expressed in >10% of the cell lines (SEMA4C, FGFRL1, PKD1, FAM38A, WDR81, TMEM136, SLC36A1, SLC26A6, IGFLR1). Analysis of these genes uncovered important roles for FGF and SEMA4 signaling in colorectal cancer with possible therapeutic implications. We also found that cell lines express on average 11 druggable mutations, including frequent mutations (>20%) in the receptor tyrosine kinases AXL and EPHA2, which have not been previously considered as potential targets for colorectal cancer. Finally, we identified 82 cell surface mutated epitopes, however expression of only 30% of these epitopes was detected in our cell lines. Notwithstanding, 92% of these epitopes were expressed in cell lines with the mutator phenotype, opening new venues for the use of “general” immune checkpoint drugs in this subset of patients.

## INTRODUCTION

Colorectal cancer is the most common gastrointestinal cancer in the world, with approximately one million new cases being diagnosed and more than 500,000 deaths occurring yearly. Approximately, one in five patients is diagnosed with metastatic disease, and an additional 30%–40% develop metastasis during the course of their disease. Unfortunately, only a minority of the patients with metastatic disease is amenable to curative resection and remains free of disease recurrence [1]. Even though survival for patients with unresectable metastatic colorectal cancer has improved over the past decade, due to the introduction of agents targeting the Epidermal Growth Factor Receptor (EGFR) and the Vascular Endothelial Growth Factor (VEGF), these treatments are often not curative, and intrinsic and acquired drug resistance is frequently observed in the clinical practice [2]. Therefore, the identification of altered pathways and new therapeutic targets is critical to improve the management of a significant proportion of colorectal cancer patients.

Genetic analysis of colorectal tumors over the past 30 years allowed the characterization of distinct molecular pathways altered during the development and progression of this disease [3]. Initial whole-exome screenings using colorectal cancer cell lines detected an average of 80 point mutations in coding regions of the genome and a small number of frequently mutated cancer genes [4]. More recently, in a major effort to dissect the genetic basis of colorectal cancer, the TCGA released the results of a comprehensive and integrated genome-scale analysis of 276 tumors. No significant genetic differences were observed between rectal and colon tumors, and twenty–four genes were identified as frequently mutated in colorectal cancer, including several novel cancer genes such as SOX9, ARID1A, ATM, TCF7L2 and FAM123B. Most importantly, new potentially druggable targets were identified, including amplifications in the ERBB2 and IGF2 genes [5]. Despite this massive sequencing effort, a recent mutation saturation analysis of 4,742 tumors, across 21 cancer types, revealed that the cancer gene catalogue is far from complete, and that many more mutated genes with putative druggable mutations remain to be discovered [6].

Cell surface proteins are involved in a variety of cellular functions, including nutrient and ion transport, adhesion and signaling. These proteins also play important roles in pathological conditions such as diabetes, neurological disorders and cancer. They represent approximately 18% of all protein-coding genes in the human genome [7] and, due to their accessibility on the cell surface, they constitute optimal targets for directed therapies [8]. We have recently generated a catalog of genes coding for transmembrane proteins located at the surface of human cells (Surfaceome), and by integrating publically available gene expression data from a variety of sources, we searched for altered pathways, new therapeutic targets and tumor antigens in gliomas, colorectal and breast tumors [9, 10]. In the present work, we carried out a systematic mutational analysis of the Surfaceome in a panel of 23 representative colorectal cancer cell lines, searching for novel altered pathways, druggable mutations and mutated epitopes for targeted therapy in colorectal cancer. Collectively, our results point towards the potential use of FDA (U.S. Food and Drug Administration) approved RTK inhibitors and immune checkpoint target drugs in specific subsets of colorectal cancer patients.

## RESULTS

### Targeted sequencing the Surfaceome in colorectal cancer

We have recently used a combined bioinformatics approach to generate a catalog of genes coding for transmembrane proteins located on the surface of human cells [9]. Briefly, we searched the complete set of protein-coding genes for an annotated and/or predicted transmembrane domain and eliminated false positive candidates containing a signal peptide or known to be located on the membrane of other intracellular compartments. An updated list of genes coding for cell surface proteins was generated for this study (Supplementary information Table S1).

To define the mutational profile of the Surfaceome in colorectal cancer, we target sequenced the coding regions of the 3,594 cell surface protein genes in a panel of 23 tumor cell lines (Supplementary information Table S2) that altogether are representative of the main subtypes of primary colorectal tumors at the genomic level [11]. A total of 33,405 exons, covering ~6Mb of the human genome, were screened for the presence of somatic point mutations (nucleotide non-synonymous substitutions and InDels). For each cell line we analyzed approximately 1.2 Gb of on target sequences, with an average coverage of 30X (Table 1).

**Table 1:** Sequencing and coverage data of the Surfaceome in colorectal cancer cell lines

Cell lines	Sequenced bases on target	Targeted region coverage	% of the target region covered	% of the target region covered >10X
CACO2	858,621,967	21.69 x	94	72
COLO205	964,932,858	23.32 x	94	76
COLO320	1,422,843,956	37.44 x	97	79
HCC2998	776,194,619	19.59 x	92	68
HCT116	865,230,723	19.63 x	87	71
HCT15	883,598,719	22.35 x	91	74
HT29	834,430,884	19.06 x	89	64
KM12	800,347,001	19.91 x	88	71
LIM1215	780,155,130	20.64 x	88	71
LIM2405	826,244,327	20.01 x	90	68
LOVO	1,484,687,708	34.87 x	97	81
RKO	826,244,327	20.34 x	92	68
RW2982	1,481,300,560	39.87 x	97	76
RW7213	1,609,474,378	43.33 x	97	78
SKCO1	1,564,643,937	41.67 x	97	81
SW1116	1,668,214,550	44.23 x	97	80
SW403	1,980,147,215	49.36 x	97	85
SW48	1,816,359,174	45.51 x	97	85
SW480	828,577,905	21.45 x	90	73
SW620	870,322,345	20.79 x	88	71
SW837	1,753,017,836	44.74 x	97	85
SW948	710,553,204	18.46 x	96	51
T84	1,761,549,149	43.46 x	97	86

### Somatic mutations in the colorectal cancer Surfaceome

**Figure 1:**
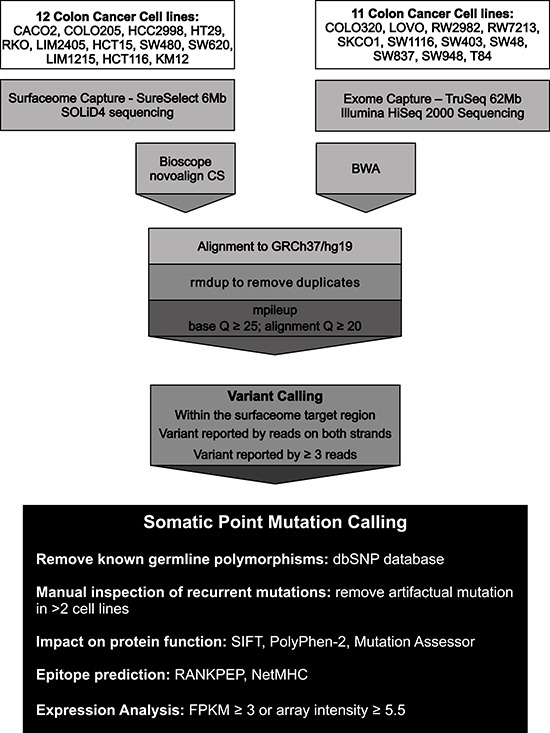
Sequencing strategy and computational pipeline used for the detection of somatic point mutations in the Surfaceome of colorectal cancer cell lines The coding regions of 3,594 cell surface proteins were screened for the presence of somatic point mutations in 23 colorectal cancer cell lines. Genomic sequences were generated using either a SOLiD4 or a HiSeq 2000 sequencing platform. Sequences were aligned against the human genome reference sequence (GRCh37/hg19) using Bioscope and NovoAlign CS for SOLiD4 sequences and BWA for HiSeq 2000 sequences. Variant calling was performed using samtools mpileup and requiring at least 3 high quality reads (Q≥25; q≥20) on both strands supporting the variant. Known germline polymorphisms were removed and recurrent mutations were manually inspected to remove alignment artifacts. SIFT, PolyPhen-2 and Mutation Assessor were used to predict the functional impact of non-synonymous substitutions on protein function. RANKPEP and NetMHC were used for epitope prediction. Gene expression data was obtained from RNASeq (FPKM>3) or microarray (hybridization intensity≥5.5) experiments.

Somatic point mutations were detected using an in house computational pipeline based on SAMtools mpileup calling (Figure 1). As matched normal tissue for these cell lines was not available, putative somatic mutations were identified by annotation against databases of known human germline variants (Table 2). A total of 3,944 putative somatic non-synonymous substitutions and 595 InDels were catalogued affecting 2,061 (57%) Surfaceome genes (Supplementary information Table S3). We identified an average of 174 putative non-synonymous substitutions and 28 InDels per cell line (Table 2). Mutation rates for genes coding for cell surface proteins varied significantly across cell lines and were similar to those previously reported for the entire set of protein-coding genes in colorectal tumors (Table 2) [5]. As expected, higher mutation rates (mutator phenotype) were observed in cell lines with microsatellite instability (MSI) and mutations in the DNA mismatch-repair genes or POLε (Supplementary information Table S2).

**Table 2:** SNV and InDel calling in the surfaceome of colorectal cancer cell lines

Cell line	SNVs	% of SNVs in dbSNP	Somatic SNVs	Somatic non-synonymous SNVs	Somatic nonsense mutations	InDels	% of InDels in dbSNP	Somatic InDels	Somatic Frameshift InDels	Mutation rate	Mutator Phenotype
CACO2	2572	98	41	24	-	51	33	12	8	6.72E-06	No
COLO205	2753	98	43	33	-	64	30	15	11	7.05E-06	No
COLO320	3813	97	86	65	4	43	53	2	2	1.21E-04	No
HCC2998	3391	78	738	569	44	52	37	16	14	5.85E-05	Yes
HCT116	3193	88	357	237	10	106	18	61	54	1.76E-04	Yes
HCT15	3959	73	1071	775	34	77	26	31	27	1.08E-05	Yes
HT29	2373	96	66	49	3	49	33	9	8	6.92E-05	No
KM12	2978	86	422	284	12	117	15	74	65	2.39E-05	Yes
LIM1215	2841	95	146	103	2	81	21	41	39	3.39E-05	Yes
LIM2405	2731	92	207	148	6	91	18	53	53	7.90E-05	Yes
LOVO	4591	88	495	352	11	130	22	82	74	1.36E-05	Yes
RKO	3500	86	482	344	15	123	16	83	75	1.62E-05	Yes
RW2982	3545	97	80	57	2	32	50	8	5	1.41E-05	No
RW7213	3763	98	62	40	-	44	48	7	1	8.12E-05	No
SKCO1	4055	96	118	79	5	43	49	5	5	1.31E-05	No
SW1116	3719	97	89	64	-	47	51	6	4	1.02E-05	No
SW403	4196	94	165	123	9	48	58	4	4	1.94E-05	No
SW48	4706	88	530	365	15	177	23	96	92	1.46E-05	Yes
SW480	2440	96	83	60	3	43	28	8	8	2.71E-05	No
SW620	2535	95	99	69	3	49	29	13	9	8.69E-05	No
SW837	3912	97	62	44	3	42	50	4	3	1.02E-05	No
SW948	2820	98	56	35	2	34	53	3	1	9.18E-06	No
T84	4089	96	118	86	2	51	51	5	2	1.94E-05	No

A total of 184 (5%) putative non-synonymous substitutions were nonsense, and 529 (89%) of the InDels introduced a frame-shift alteration in the mutated protein (Table 2 and Supplementary information Table S4). To further identify substitutions that may impact protein function, we used three different algorithms (PolyPhen, SIFT and Mutation Assessor) to estimate the impact of amino acid substitutions using information from DNA sequence, evolutionary conservation and structural data. A total of 1,434 (36%) putative non-synonymous substitutions and 474 (80%) InDels were classified as having an impact on protein function, and colorectal cancer cell lines harbor on average 85 putative point mutations (non-synonymous substitutions and indels) with a predicted impact on protein function (Table 3 and Supplementary information Table S5).

**Table 3:** Analysis of somatic point mutations present in the surfaceome of colorectal cancer cell lines

Cell line	Mutator phenotype	Non-synonymous mutations with predicted functional impact	InDels with predicted functional impact	Druggable mutations	Expressed druggable mutations	Mutated epitopes	Expressed mutated epitopes
CACO2	No	8	6	4	3	-	-
COLO205	No	10	11	9	2	1	0
COLO320	No	24	1	15	3	2	0
HCC2998	Yes	196	9	128	17	11	3
HCT116	Yes	81	45	66	19	7	4
HCT15	Yes	287	20	184	57	19	7
HT29	No	10	4	16	1	2	1
KM12	Yes	91	54	74	27	6	2
LIM1215	Yes	42	33	40	13	-	-
LIM2405	Yes	48	46	56	16	7	2
LOVO	Yes	160	57	69	19	8	2
RKO	Yes	131	72	95	30	7	2
RW2982	No	17	3	9	2	3	0
RW7213	No	13	4	10	2	-	-
SKCO1	No	24	3	10	3	1	0
SW1116	No	29	3	9	3	2	1
SW403	No	36	4	22	4	-	-
SW48	Yes	141	75	91	24	4	2
SW480	No	26	7	13	7	3	1
SW620	No	26	11	18	4	1	0
SW837	No	9	2	6	2	1	0
SW948	No	14	3	8	1	-	-
T84	No	27	4	15	3	1	0

### Novel mutated cell surface proteins and altered pathways in colorectal cancer

To further address the biological significance of the uncovered point mutations, we have incorporated gene expression data available for the cell lines (RNAseq and microarray) and restricted our downstream analysis to mutated and expressed genes. A list of genes coding for cell surface proteins that are mutated and expressed in >10% of the 23 cell lines analyzed is provided in Supplementary information Table S6. Analysis of expressed mutated surface genes revealed recurrent mutations in genes belonging to pathways known to be involved in colorectal cancer, including the WNT (LRP5 and FZD10), TGFβ (TGFBR3 and ACVR1B) and RTK-Ras (EGFR and ERBB3) signaling pathways [5]. Our analysis also identified 48 expressed genes that were not previously described as mutated in primary colorectal tumors in the TCGA database [5] (Supplementary information Table S7). This list includes mutations in 9 genes (SEMA4C, FGFRL1, PKD1, FAM38A, WDR81, TMEM136, SLC36A1, SLC26A6, IGFLR1) that occur in >10% of the cell lines and were confirmed by Sanger sequencing.

**Figure 2:**
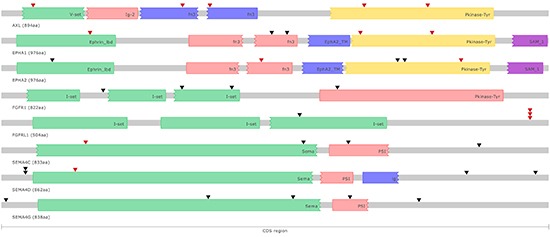
Schematic representation of somatic point mutations affecting the coding regions of putative druggable genes in colorectal cancer cell lines Known protein domains are represented using different colors. Somatic point mutations occurring in different colorectal cancer cell lines are indicated (▼) and highlighted in red when predicted to have an impact on protein function.

Semaphorin 4C (SEMA4C) mutations were detected and validated by Sanger sequencing in 4 cell lines (HCT15, KM12, RW2982, T84). Two of these mutations occur in the SEMA domain, a highly conserved sequence of approximately 500 amino acids critical for inducing targets of Semaphorin signaling. A third mutation occurs in the plexin-semaphorin-integrin (PSI) domain, another highly conserved domain, enriched in cysteine residues (Figure 2). Recurrent mutations in other genes belonging to the Semaphorin signaling pathway were also observed, including frequent mutations (>20%) in SEMA4G and SEMA4D, some of which also occurring in the SEMA and PSI domains (Figure 2). Semaphorins are an evolutionarily conserved family of proteins that have been initially implicated in nervous system development and, more recently, in cancer progression and tumor angiogenesis [12, 13]. SEMA4C expression is significantly down-regulated during stem cell differentiation [14] and plays an important role in TGFβ-1 induced epithelial-mesenchymal transition [15]. To date, there is no published evidence of the direct involvement of SEMA4C in cancer, but somatic point mutations in SEMA4C were also reported by TCGA in 4% of the cutaneous melanomas. Conversely, an important role of the SEMA4D-Plexin-B1 interaction in regulating different aspects of tumor progression and angiogenesis is well established [16]. In all, alterations in SEMA4 family members were detected in 56% (13/23) of the cell lines, indicating an important role of SEMA4 signaling in colorectal cancer.

Fibroblast Growth Factor Receptor Like Protein 1 (FGFRL1) alterations were detected and validated by Sanger sequencing in 4 cell lines (LOVO, KM12, LIM1215, RKO). Three cell lines carry frameshift mutations and the remaining cell line carries a non-synonymous point mutation with a predicted damaging effect, indicating a loss of function of the FGFRL1 protein in colorectal cancer (Figure 2). Recurrent (>10%) FGFRL1 somatic mutations were also reported in bladder tumors [17]. FGFRL1 acts as a negative regulator of Fibroblast Growth Factor Receptor 1 (FGFR1) signaling either by interfering with FGFR1 dimerization and phosphorylation or by sequestering FGFR1 ligands [18]. FGFR1 amplification and overexpression has been reported in colorectal cancer and associated with the presence of liver metastasis [19]. Indeed, in our study we also detected and validated by Sanger sequencing somatic mutations in FGFR1 in 4 cell lines (HCT116, HCT15, RKO, SW48), including a non-synonymous substitution in the tyrosine kinase domain (Figure 2). Mutations in FGFR2 (LIM2405) and FGFR3 (LOVO, SW48) were also observed at a lower frequency. In all, alterations in FGFR family members were detected in 35% (8/23) of the cell lines, suggesting an important role of FGF signaling in colorectal cancer.

Although the remaining 7 genes (PKD1, FAM38A, WDR81, TMEM136, SLC36A1, SLC26A6, IGFLR1) are mutated in >10% of the colorectal cancer cell lines, literature searches did not reveal evidence of the functional role or therapeutic potential of these genes in colorectal cancer. Nevertheless, recurrent mutations (>3%) in FAM38A, SLC36A1 and WDR81 have been reported for other primary tumors in the TCGA database, and further functional studies will be necessary to address their involvement in colorectal tumorigenesis.

### Druggable mutations in cell surface proteins for targeted therapy in colorectal cancer

In order to identify putative druggable mutations in cell surface proteins, we searched for mutated genes present in the Drug-Gene Interaction Database (DGIdb), which integrates drug-target information from 13 different sources, including the literature and previously established databases [20]. We generated a catalogue of point mutations in druggable genes, and we found that colorectal cell lines harbor on average 11 mutations in druggable expressed genes (Table 3 and Supplementary information Table S8).

A significant fraction (34%) of these mutations occurred in membrane transporters. Membrane transporters, including solute carriers (SLCs) and ABC transporters, control the uptake and efflux of amino acids, sugars, lipids and vitamins, and their expression and activity are frequently altered in cancer as a consequence of the higher energy and nutritional requirements of the tumor cells [21]. Membrane transporters represent potential targets for cancer therapy and blocking their activity could be one way to interfere with tumor progression. In addition, membrane transporters can also serve as chemo-sensitizing targets, since they actively participate in drug delivery and resistance [21, 22]. Mutations in ABCA3, ABCA7, ABCC1, SLC23A2 and SLC9A1 were each observed in >20% of the cell lines.

We then focused on expressed genes with putative druggable mutations that were not previously considered as potential therapeutic targets for colorectal cancer, but for which specific inhibitors have previously been developed. We particularly focused on surface proteins with kinase activity, as they represent a significant fraction of the genes mutated in cancer and are highly amenable to targeting by rationally designed small molecule inhibitors. Two druggable RTKs (AXL and EPHA2) were found to be frequently (>20%) altered in our cell lines.

Five point mutations in the kinase domain and/or with predicted functional impact in the AXL Receptor Tyrosine Kinase (AXL) were detected and validated by Sanger Sequencing in 22% (5/23) of our cell lines (COLO205, KM12, HCT116, HCT15 and LOVO) (Figure 2). One of these mutations (g.chr19:41726597 C>T) occurring in the GAS6-ligand binding domain was also observed in a uterine corpus endometrioid carcinoma and in a glioblastoma. AXL is a member of the TAM family of RTKs, which also includes Mer and Tyro-3 [23]. Mutations in Mer (SW48) and Tyro-3 (HCT15) were also observed. Point mutations in AXL have not been specifically described in the literature for colorectal cancer, and lower mutation frequencies (3.5%) were reported for primary colorectal cancers in the TCGA database [5]. Overexpression of AXL in colorectal tumors was reported in metastatic lesions [24] and AXL was recently characterized as a poor prognostic marker in early stage colorectal tumors, and as an important mediator of basal and 5-FU induced EMT and invasiveness [25].

Point mutations in the EPH Receptor A2 gene (EPHA2) were detected and validated by Sanger sequencing in 3 cell lines (HCT15, LIM1215, LIM2405). Three of these mutations are located in the tyrosine kinase domain and one in the Ephrin-ligand binding domain (Figure 2). Mutations with predicted functional impact in the Ephrin-ligand binding domain and in the tyrosine kinase domain of the EPHA1 gene were also observed in 3 cell lines (HCT15, LIM1215 and LOVO) (Figure 2). Point mutations in EPHA2 and EPHA1 have not been specifically described in the literature for primary colorectal tumors, and lower mutation frequencies for these genes (4.4% EPHA1 and 2.6% EPHA2) were reported for primary colorectal tumors in the TCGA database [5]. EPHA2 is overexpressed in tumor cells and in tumor blood vessels in different types of cancer [26]. In colorectal tumors, EPHA2 overexpression was detected in approximately half of the samples and higher expression was associated with advanced stage tumors, metastatic disease and higher microvessels counts [27, 28]. Moreover, loss of EPHA2 reduced tumor formation in Apc Min/+ mice [29]. Conversely, elevated levels of EPHA1 were observed in early stage compared to late stage colorectal tumors. Reduced EPHA1 expression was associated with poorly differentiated and invasive tumors and poor overall survival, indicating that EPHA1 may play different roles during different stages of colorectal carcinoma progression [30, 31].

### Mutated epitopes exposed on the cell surface of colorectal cancer

Non-synonymous and frameshift mutations in the Surfaceome of the 23 colorectal cancer cell lines were used to identify mutated epitopes with differential binding affinity to HLA when compared to epitopes generated by the corresponding non-mutated (reference) sequences. Our local pipeline for immunogenic epitope prediction was based on two algorithms RANKPEP and NetMHC as described in Materials and Methods. Mutated epitopes were required to have a binding affinity to the HLA*0201 molecule that was at least 20% higher than the reference epitope as predicted by both algorithms. A total of 82 putative mutated epitopes were identified (73 epitopes from non-synonymous mutations and 9 epitopes from frameshift mutations). However, when we combined gene expression data with epitope prediction analysis, we found that only 30% (25/82) of the predicted epitopes are expressed, and that 92% (23/25) of these epitopes are expressed in a subset of the cell lines with the mutator phenotype. These results suggest that the use of potentially immunogenic mutations in cell surface proteins for personalized T-cell based immunotherapy in colorectal cancer is limited, as only 30% of the mutated epitopes are expressed and less than half (11/23) of the tumors cell lines express mutated epitopes.

### Discussion and Therapeutic Implications

One of the major objectives of cancer genome sequencing projects is to identify therapeutically targetable mutations. This objective has been achieved with repeated success in cancer therapy, resulting in the introduction of new treatment protocols in the clinical practice. The use of Imatinib, for chronic myeloid leukemia and other solid tumors, of Trastuzumab and Lapatinib, for ERBB2 positive breast cancer, and of Vemurafenib, for BRAF mutant melanomas, are emblematic examples of how genomic alterations can be used to target cancer cells [32]. Over the past years, these sequencing projects have revealed many new cancer genes, most of which are mutated at intermediate frequencies (2–20%) or lower, uncovering an unprecedented level of genetic heterogeneity in human cancers and establishing the need for a continued effort to determine the functional significance of these mutations and to translate these findings to the bedside [33].

Cell surface proteins constitute optimal targets for directed therapies and represent two-thirds of the protein-based drug targets [34, 35]. Surface proteins are also excellent targets for antibody-based therapies and vaccine development since they are exposed on the cell surface and, therefore, have the highest chances to be recognized as antigens [36]. In the present work, we carried out a systematic mutational analysis of human genes coding for cell surface proteins, aiming to uncover novel altered pathways, druggable mutations and mutated epitopes for targeted therapy in colorectal cancer. We target sequenced the coding regions of cell surface protein genes in a panel of 23 tumor cell lines that altogether are representative of the main subtypes of primary colorectal tumors at the genomic level [11]. We opted to use cell lines in this study, instead of primary tumors, to overcome limitations imposed by the high level of colorectal intratumoral genetic heterogeneity in the mutation detection efficiency and to have straightforward cell models to further address the therapeutic potential of the uncovered altered pathways and druggable mutations.

We found that a significant (57%) fraction of the Surfaceome is reshaped by somatic point mutations in colorectal cancer cell lines. Our analysis identified 48 genes coding for cell surface proteins that were not previously described as mutated in primary colorectal tumors in the TCGA database [5], including mutations in SEMA4C and FGFRL1 which have not been previously considered as potential therapeutic targets for colorectal cancer. Although we cannot exclude the possibility that some of these alterations correspond to mutations acquired during *in vitro* propagation of the cell lines, our results are in agreement with a recent mutation saturation analysis of 4,742 sequenced tumors, across 21 cancer types [6]. This study revealed that the discovery of cancer genes mutated at frequencies of 5–10% in colorectal tumors is increasing linearly in relation to the number of tumor genomes sequenced, and that the current collection of sequenced colorectal tumors lacks the desired power to detect genes mutated at frequencies of 5% above the background rate [6].

SEMA4C mutations were found in 17% of the cell lines and recurrent mutations in SEMA4G (17%) and SEMA4D (22%) were also observed. The effects of Semaphorins and their receptors in cancer are broad, context dependent and complex [37]. SEMA4C is expressed in neural stem cells and its expression is downregulated during stem cell differentiation [14]. SEMA4C expression is induced by TGFβ-1 in renal epithelial cells and plays and important role in TGFβ-1 induced epithelial-mesenchymal transition [15]. In addition, an important role of SEMA4D-Plexin-B1 interaction in regulating different aspects leading to tumor progression, including invasive growth and angiogenesis, is well established [16]. The pro-angiogenic effect of SEMA4D was demonstrated both *in vitro* and *in vivo* and is comparable to that elicited by other well-known angiogenic molecules, such as VEGF-A, HGF and bFGF [38, 39]. Our results suggest that SEMA4 signaling is activated by point mutations in a significant fraction of colorectal tumors, and although specific inhibitors targeting SEMA4 proteins are not currently available, several biological process driven by SEMA4 signaling, such as angiogenesis and invasiveness, could be targeted with FDA approved drugs, including anti-angiogenic agents and MET inhibitors.

Inactivating mutations in FGFRL1, the most recently discovered member of the FGFR family, were detected in 17% of our cell lines. FGFRL1 binds with high affinity to heparin and FGF ligands, but it does not possess an intracellular protein kinase domain and, therefore, cannot signal by trans-auto-phosphorylation [18]. FGFRL1 thus acts as a negative regulator of FGFR1 signaling and loss of function mutations described here may represent a novel mechanism of FGF signaling activation in colorectal cancer. Alterations in FGFR1, FGFR2 and FGFR3 were also observed at a lower frequency, and 35% of the cell lines harbored somatic mutations in members of the FGF signaling pathway. Different FGFR specific inhibitors are currently under development [40], and further evaluation of their activity in the subset of colorectal cancer with FGFR/FGFRL1 alterations should be pursued. Moreover, Regorafenib, a multi-kinase inhibitor that targets FGFR1 among other RTKs, was recently approved by the FDA for the treatment of advanced colorectal cancer [41], but predictive biomarkers for this indication are not yet currently available.

Higher mutation frequencies in the RTKs AXL (22%) and EPHA2 (17%) were detected in our panel compared to those reported in the TCGA database for primary colorectal tumors (3.51% AXL and 2.63% EPHA2) [5]. Both RTKs have not been considered as potential therapeutic targets for colorectal cancer, however the availability of specific inhibitors and pre-clinical data support their potential use for therapeutic intervention. The oncogenic properties of AXL were initially described in patients with chronic myelogenous and lymphoblastic leukemia (CML), but overexpression of AXL have also been detected in many solid tumors and associated with poor prognosis [23]. AXL has a well established oncogenic role in survival, proliferation and migration of cancer cells *in vitro*, as well as in tumor angiogenesis and metastasis *in vivo* [23]. Moreover, recent studies have uncovered a major role of AXL in primary and acquired resistance to several anticancer therapies. AXL overexpression has been linked to Imatinib-resistance in gastrointestinal stromal tumors [42], Nilotinib-resistance in CML [43] and Lapatinib-resistance in HER-2 positive breast tumor cells [44]. In lung cancer, AXL was identified as a potential target for overcoming EGFR inhibitor resistance and combination of an AXL specific inhibitor (SGI-7079) with Erlotinib reversed Erlotinib resistance in a xenograft model of mesenchymal non-small cell lung cancer [45].

In colorectal cancer, AXL expression is associated to increased invasiveness of tumor cell lines with overexpression of the chemokine receptors CXCR4 and CXCR7, and AXL knock-down in these cell lines significantly hampered tumor cell invasion [46]. Considering that many multi-kinase inhibitors under development have AXL as one of their targets, further exploration of the pharmacologic inhibition of this pathway in pre-clinical models, including tumor cells lines with resistance to anti-EGFR drugs, should be pursued. In addition, monoclonal antibodies and small-molecule tyrosine kinase inhibitors specifically targeting AXL are currently in development and their use in colorectal cancer patients should also be further explored [47]. Noteworthy, some of the cell lines analyzed herein presented concomitant mutations in AXL and FGFR or FGFRL1 (HCT116, HCT15, LOVO, KM12), which suggests that these mutations are not mutually exclusive. In this setting, it will be important to explore the interdependence of both pathways, specially considering that some multi-kinase inhibitors under development are capable of blocking AXL and FGFR concomitantly [48]. Indeed, combination of these multi-kinase inhibitors with bevacizumab led to near total inhibition of tumor growth in colon carcinoma xenograft models and caused tumor growth arrest in bevacizumab-resistant tumors [48].

Somatic alterations in EPH receptors were also frequently observed in our cell lines, including frequent mutations in EPHA1 and EPHA2. Point mutations in EPHA2 and EPHA1 have not so far been described in the literature for colorectal cancer. Nevertheless, mutations in the kinase domain of EPHA3 was reported in 5% of colorectal cancer cell lines [49] and EPHA3 was listed among the top 3 cancer genes in a large-scale screening for somatic mutations in colorectal cancer [4]. EPH receptors play critical roles in embryonic development and their expression is frequently altered in a variety of cancers and tumor cell lines [50]. They comprise the largest family of RTKs and bind to ephrins (EFN) available on the surface of neighboring cells. Unlike others RTKs, EPH-EFN signaling is unique, since it triggers a bi-directional signal that affects both receptor and EFN expressing cells [50]. EPH receptors are thus important mediators of tumor cell interactions with the tumor stroma and tumor vasculature, and have been proposed as promising targets for cancer therapy, since targeting these receptors could simultaneously inhibit several aspects of tumor progression [26, 50]. EPHA2 overexpression in colorectal cancer is associated with advanced stage tumors, metastatic disease and higher microvessel counts [27, 28]. Moreover, loss of EPHA2 was shown to reduce Apc Min/+ tumorigenesis [29]. Confirmation of the activation of EPH signaling mediated by EPHA2 point mutations in colorectal cancer is of upmost importance considering the availability of FDA approved drugs targeting this receptor, such as Dasatinib [51]. In addition, EPHA2-FC soluble receptors were shown to significantly reduce tumor volume and overall metastatic burden in pre-clinical models of breast [52] and pancreatic tumors [53], but have not been evaluated in colorectal cancer models. Finally, receptor endocytosis promoted by anti-EPHA2 monoclonal antibodies has also been used to reduce EPHA2 activity and inhibit malignant cell behavior *in vitro* [54]. On the other hand, therapies targeting EPHA1 in colorectal cancer should be carefully evaluated since this gene seems to play different roles during disease progression [30, 31].

Non-synonymous and frameshift mutations in tumor cells can generate unique T-cell mutated epitopes and induce tumor antigen-specific immune response [55]. There is evidence supporting the efficacy of vaccination strategies using mutated epitopes [56] and the use of personalized peptide vaccines and adoptive T-cell transfer protocols based on patient-specific mutated epitopes holds great promise in cancer therapy [57]. Unfortunately, combining epitope prediction algorithms and gene expression data, we found that the use of potentially immunogenic mutations in cell surface proteins for personalized immunotherapy in colorectal cancer is limited, since the expression of approximately 70% of these epitopes was not detected in the tumor cells. However, additional studies including mutated epitopes present in intracellular proteins will be required to further address the applicability of personalized vaccines in colorectal patients.

Notwithstanding, we observed that mutated expressed epitopes are predominantly found in colorectal cell lines presenting a mutator phenotype and that this specific subset of cell lines express a total of 23 mutated epitopes. In this context, it was recently demonstrated that patients with tumors showing naturally occurring immunogenic mutations presented higher cytotoxic T-cell infiltration and improved overall survival and, based on these observations, the use general immune modulators that block immune regulatory checkpoints such as anti-CTLA4 and anti-PD1 was proposed as a treatment strategy for patients with immunogenic mutations [58]. Accordingly, tumors with a high level of mutations as revealed by the TCGA [59], such as melanoma and non-small cell lung cancer, are currently deriving striking benefits with immune checkpoint blockage drugs [60, 61]. Although our results do not support the use of personalized T-cell based immunotherapy in colorectal cancer, they suggest that colorectal cancer patients harboring tumors with a mutator phenotype could be more responsive to immune checkpoint blockage. Indeed, increased counts of CD8+ T-cells were observed in colorectal cancer tumors with high mutational loads [58] and microsatellite instability [62]. Data on the use of immune checkpoint target drugs in colorectal cancer are still limited, but the results of the first long term follow-up study from the first clinical trial based on the PD1-targeting monoclonal antibody have recently been reported. This study included a 71-year-old patient with colorectal cancer who attained a complete and durable (>4 years) response to anti-PD1 treatment [63].

To the best of our knowledge, this is the first systematic and focused screen of point mutations in genes coding for cell surface proteins in colorectal cancer. By combining high-throughput sequencing, bioinformatics tools, data integration and literature searches, we have successfully discovered novel altered pathways and druggable mutations for targeted therapy in colorectal cancer. We have also uncovered the potential use of existing RTK inhibitors and immune checkpoint target drugs in specific subsets of colorectal cancer patients. Results presented here are encouraging, however our study also presents some limitations.

First, although we have described novel druggable mutations occurring in a representative panel of colorectal cancer cell lines, it will be important to confirm the prevalence of these alterations in clinical samples matched with normal tissue. At present, we cannot completely exclude the possibility that some of the alterations reported in this study correspond to mutations acquired during *in vitro* propagation of the cell lines or to very rare germline polymorphisms not represented in public databases, nor in individuals sequenced by the 1000 Genomes Project. However, we believe that these possibilities do not significantly affect our results, since we have previously shown that the rate of mutation accumulation during *in vitro* propagation is not significant [11] and stringent bioinformatics cut-offs were implemented to filter most, if not all, non-clonal mutations eventually introduced during *in vitro* growth. Second, the functional consequences of the uncovered genetic alterations were predicted primarily using computational tools, and confirmation with functional *in vitro* assays is further required. Similarly, additional experiments to evaluate the effects of pharmacologic inhibition of the altered pathways using pre-clinical models are compulsory to translate our findings to the bedside. Finally, although we suggest potential molecular therapeutic targets in colon cancer, it is important to recognize that a recent study matching targeted therapy with specific molecular abnormalities for patients with advanced colorectal cancer failed to confer significant clinical benefit [64]. We believe that a diversification of potential targets, including those proposed by our study, could bring new opportunities to change this paradigm.

## MATERIALS AND METHODS

### Colorectal cancer cell lines

The panel of 23 colorectal cancer cell lines used in this study was obtained from different sources (Supplementary information Table S2). CACO2, COLO205, COLO320-DM, HCT116, HCT15, HT29, LOVO, RKO, SKCO-1, SW1116, SW403, SW48, SW480, SW620, SW837, SW948 and T84 were obtained from the American Type Culture Collection (Manassas, VA). LIM1215 and LIM2405 were generated by the Ludwig Institute for Cancer Research. HCC2998 and KM12 were obtained from the National Cancer Institute-Frederick Cancer DCT Tumor Repository. RW2982 and RW7213 were provided by Dr. P Calabresi from Roger Williams General Hospital. Cells were cultured with Dulbecco's Modified Eagle Medium and 10% FBS at 37^o^C and 5% CO_2_. Cell lines were authenticated and tested for *Mycoplasma* contamination as previously described [11].

### Target sequencing the human surfaceome

We target-sequenced the coding regions of 3,594 cell surface proteins in 12 cell lines (CACO2, COLO205, HCT116, HCT15, HT29, RKO, SW480, SW620, LIM1215, LIM2405, HCC2998, KM12). Surfaceome-capture and sequencing were performed using Sure Select Target Enrichment baits (Agilent Technologies) and the SOLiD 4.0 sequencing platform (Life Technologies), respectively. For the remaining cell lines (COLO320, LOVO, SKCO1, SW1116, SW403, SW48, SW837, SW948, T84, RW2982 and RW7213) whole-exome capture was performed using the TruSeq Exome Enrichment Kit (Illumina) and paired-end sequencing was performed using Illumina HiSeq 2000. A local pipeline was then developed to extract the genomic sequences corresponding to the Surfaceome targeted region from whole-exome data.

### Public Data and Databases

Exome-capture sequencing data on colorectal tumors were retrieved from TCGA and used to identify novel mutated genes and to determine mutation frequencies in colorectal cancer primary tumors. The DGIdb [20] was used to identify druggable mutated genes and the gene list provided by the Human Kinome project [65] (kinase.com/human/kinome) was used to identify genes coding for cell surface proteins with kinase activity.

### Somatic mutation detection, validation and functional analysis

For single nucleotide variations (SNVs) detection, SOLiD 4.0 and Illumina reads were aligned to the human reference genome sequence (GRCh37/hg19) using BioScope (Life Technologies) and BWA [66], respectively. For InDels detection, alignments were performed using NovoAlignCS (www.novocraft.com). A local pipeline for point mutations was developed using Samtools mpileup and bcftools [67]. Duplicated reads were removed with rmdup (Samtools) to avoid potential PCR duplicates generated during library construction. Variants were filtered against known germline variations annotated in dbSNP (version #135) and variations present in more than 3 cell lines were manually inspected to distinguish recurrent mutations (eg. EGFR mutations) from false positive mutations due to alignment artifacts. Somatic mutations were validated using PCR amplification and Sanger sequencing using standard protocols (Supplementary information Table S9, S10). SIFT [68], PolyPhen-2 [69] and Mutation Assessor [70] were used to evaluate the impact of non-synonymous substitutions and InDels on protein function. Mutations were annotated as having an impact on protein function when predicted by at least two of these algorithms in the case of non-synonymous substitutions and by SIFT in the case of InDels.

### Gene expression data

RNA-Seq data was generated for 12 cell lines (CACO2, COLO205, COLO320, HCT116, HCT15, HT29, KM12, LIM1215, LIM2405, RKO, SW480, SW948) using the 5500XL sequencing platform to a depth of >100 million reads. Sequences were aligned to the human reference genome sequence (GRCh37/hg19) using TopHat [71] with standard parameters for color space reads. Isoform assembly and transcript relative abundance was determined using Cufflinks [72]. Genes were considered expressed when FPKM [72, 73] was ≥ 3 in at least one of the cell lines [73, 74]. For the remaining cell lines (LOVO, SKCO1, SW1116, SW403, SW48, SW620, SW837, e T84) microarray expression data was extracted from GEO [75]; Accession GSE36133) and genes were considered expressed when the array values were ≥ 5.5.

### Epitope prediction

Peptide sequences corresponding to non-synonymous mutations and InDels, flanked by 10 aminoacids on either side, were used for epitope prediction by applying a similar approach to that described by Segal et al. 2008 [76]. The same process was performed for peptide sequences corresponding to the non-altered (reference) sequences. Concatamers of these peptides were analyzed by RANKPEP [77] and NetMHC [78] to identify 9 aa peptide sequences with binding affinity to the class I MHC molecule HLA-A*0201. RANKPEP predicts binding based on scoring matrices from known peptides that bind to MHC molecules. Peptides were considered immunogenic if the percentage optimum was ≥ 50%. RANKPEP also evaluates if the peptide tested results from a known cleavage process and therefore only predicted cleaved peptides were analyzed. NetMHC uses artificial neural networks to predict binding to the MHC molecule. The peptides were considered immunogenic if the IC_50_ was ≤ 500nM. To check for predicted cleavage, sequences were then analyzed using the NetChop algorithm [79], and only peptides with predicted cleavage were selected. Results from both algorithms were processed using a local pipeline and epitopes resulting from sequence concatenation artifacts were excluded. Mutated epitopes were defined as those predicted by both algorithms and that were unique to the variant sequence or showing an increase in MHC biding affinity by >20% when compared to the reference peptide.

## SUPPLEMENTARY TABLES



## References

[1] Siegel R, Desantis C, Jemal A (2014). Colorectal cancer statistics, 2014. CA. Cancer J. Clin.

[2] Arnold D, Seufferlein T (2010). Targeted treatments in colorectal cancer: state of the art and future perspectives. Gut.

[3] Fearon ER (2011). Molecular genetics of colorectal cancer. Annu. Rev. Pathol.

[4] Sjöblom T, Jones S, Wood LD, Parsons DW, Lin J, Barber TD, Mandelker D, Leary RJ, Ptak J, Silliman N, Szabo S, Buckhaults P, Farrell C (2006). The consensus coding sequences of human breast and colorectal cancers. Science.

[5] TCGA, Cancer T, Atlas G (2012). Comprehensive molecular characterization of human colon and rectal cancer. Nature.

[6] Lawrence MS, Stojanov P, Mermel CH, Robinson JT, Garraway LA, Golub TR, Meyerson M, Gabriel SB, Lander ES, Getz G (2014). Discovery and saturation analysis of cancer genes across 21 tumour types. Nature.

[7] Harrow J, Frankish A, Gonzalez JM, Tapanari E, Diekhans M, Kokocinski F, Aken BL, Barrell D, Zadissa A, Searle S, Barnes I, Bignell A, Boychenko V (2012). GENCODE: the reference human genome annotation for The ENCODE Project. Genome Res.

[8] Rask-Andersen M, Almén MS, Schiöth HB (2011). Trends in the exploitation of novel drug targets. Nat. Rev. Drug Discov.

[9] Da Cunha JP, Galante PA, de Souza JE, de Souza RF, Carvalho PM, Ohara DT, Moura RP, Oba-Shinja SM, Marie SK, Silva WA, Perez RO, Stransky B, Pieprzyk M (2009). Bioinformatics construction of the human cell surfaceome. Proc Natl Acad Sci U S A.

[10] Da Cunha JPC, Galante PAF, de Souza JES, Pieprzyk M, Carraro DM, Old LJ, Camargo AA, de Souza SJ (2013). The human cell surfaceome of breast tumors. Biomed Res. Int.

[11] Mouradov D, Sloggett C, Jorissen RN, Love CG, Li S, Burgess AW, Arango D, Strausberg RL, Buchanan D, Wormald S, O'Connor L, Wilding JL, Bicknell D (2014). Colorectal cancer cell lines are representative models of the main molecular subtypes of primary cancer. Cancer Res.

[12] Flannery E, Duman-Scheel M (2009). Semaphorins at the interface of development and cancer. Curr. Drug Targets.

[13] Gu C, Giraudo E (2013). The role of semaphorins and their receptors in vascular development and cancer. Exp. Cell Res.

[14] Wu H, Fan J, Zhu L, Liu S, Wu Y, Zhao T, Wu Y, Ding X, Fan W, Fan M (2009). Sema4C expression in neural stem/progenitor cells and in adult neurogenesis induced by cerebral ischemia. J. Mol. Neurosci.

[15] Zeng R, Han M, Luo Y, Li C, Pei G, Liao W, Bai S, Ge S, Liu X, Xu G (2011). Role of Sema4C in TGF-β1-induced mitogen-activated protein kinase activation and epithelial-mesenchymal transition in renal tubular epithelial cells. Nephrol. Dial. Transplant.

[16] Ch'ng ES, Kumanogoh A (2010). Roles of Sema4D and Plexin-B1 in tumor progression. Mol. Cancer.

[17] Guo G, Sun X, Chen C, Wu S, Huang P, Li Z, Dean M, Huang Y, Jia W, Zhou Q, Tang A, Yang Z, Li X (2013). Whole-genome and whole-exome sequencing of bladder cancer identifies frequent alterations in genes involved in sister chromatid cohesion and segregation. Nat. Genet.

[18] Trueb B (2011). Biology of FGFRL1, the fifth fibroblast growth factor receptor. Cell. Mol. Life Sci.

[19] Sato T, Oshima T, Yoshihara K, Yamamoto N, Yamada R, Nagano Y, Fujii S, Kunisaki C, Shiozawa M, Akaike M, Rino Y, Tanaka K, Masuda M (2009). Overexpression of the fibroblast growth factor receptor-1 gene correlates with liver metastasis in colorectal cancer. Oncol. Rep.

[20] Griffith M, Griffith OL, Coffman AC, Weible JV, McMichael JF, Spies NC, Koval J, Das I, Callaway MB, Eldred JM, Miller CA, Subramanian J, Govindan R (2013). DGIdb: mining the druggable genome. Nat. Methods.

[21] El-Gebali S, Bentz S, Hediger M a, Anderle P (2013). Solute carriers (SLCs) in cancer. Mol. Aspects Med.

[22] Kunická T, S P (2014). Importance of ABCC1 for cancer therapy and prognosis. Drug Metab. Rev.

[23] Paccez JD, Vogelsang M, Parker MI, Zerbini LF (2014). The receptor tyrosine kinase Axl in cancer: biological functions and therapeutic implications. Int. J. Cancer.

[24] Craven RJ, X L (1995). Receptor tyrosine kinases expressed in metastatic colon cancer. Int. J. Cancer.

[25] Dunne PD, McArt DG, Blayney JK, Kalimutho M, Greer S, Wang T, Srivastava S, Ong CW, Arthur K, Loughrey M, Redmond K, Longley DB, Salto-Tellez M (2014). AXL is a key regulator of inherent and chemotherapy-induced invasion and predicts a poor clinical outcome in early-stage colon cancer. Clin. Cancer Res.

[26] Ireton RC, Chen J (2005). EphA2 receptor tyrosine kinase as a promising target for cancer therapeutics. Curr. Cancer Drug Targets.

[27] Saito T, Masuda N, Miyazaki T, Kanoh K, Suzuki H, Shimura T, Asao T, Kuwano H (2004). Expression of EphA2 and E-cadherin in colorectal cancer: Correlation with cancer metastasis. Oncol. Rep.

[28] Kataoka H, Igarashi H, Kanamori M, Ihara M, Wang J-D, Wang Y-J, Li Z-Y, Shimamura T, Kobayashi T, Maruyama K, Nakamura T, Arai H, Kajimura M (2004). Correlation of EPHA2 overexpression with high microvessel count in human primary colorectal cancer. Cancer Sci.

[29] Bogan C, Chen J, O'Sullivan MG, Cormier RT (2009). Loss of EphA2 receptor tyrosine kinase reduces ApcMin/+ tumorigenesis. Int. J. Cancer.

[30] Herath NI, Doecke J, Spanevello MD, Leggett Ba, Boyd a W (2009). Epigenetic silencing of EphA1 expression in colorectal cancer is correlated with poor survival. Br. J. Cancer.

[31] Dong Y, Wang J, Sheng Z, Li G, Ma H, Wang X, Zhang R, Lu G, Hu Q, Sugimura H, Zhou X (2009). Downregulation of EphA1 in colorectal carcinomas correlates with invasion and metastasis. Mod. Pathol.

[32] McDermott U, Downing JR, Stratton MR (2011). Genomics and the continuum of cancer care. N. Engl. J. Med.

[33] Chang K, Creighton CJ, Davis C, Donehower L, Drummond J, Wheeler D, Ally A, Balasundaram M, Birol I, Butterfield YSN, Chu A, Chuah E, Chun H-JE (2013). The Cancer Genome Atlas Pan-Cancer analysis project. Nat. Genet.

[34] Hopkins AL, Groom CR (2002). The druggable genome. Nat. Rev. Drug Discov.

[35] Overington JP, Al-Lazikani B, Hopkins AL (2006). How many drug targets are there?. Nat. Rev. Drug Discov.

[36] Olaya-Abril A, Jiménez-Munguía I, Gómez-Gascón L, Rodríguez-Ortega MJ (2014). Surfomics: shaving live organisms for a fast proteomic identification of surface proteins. J. Proteomics.

[37] Rehman M, Tamagnone L (2013). Semaphorins in cancer: biological mechanisms and therapeutic approaches. Semin. Cell Dev. Biol.

[38] Conrotto P, Valdembri D, Corso S, Serini G, Tamagnone L, Comoglio PM, Bussolino F, Giordano S (2005). Sema4D induces angiogenesis through Met recruitment by Plexin B1. Blood.

[39] Basile JR, Castilho RM, Williams VP, Gutkind JS (2006). Semaphorin 4D provides a link between axon guidance processes and tumor-induced angiogenesis. Proc. Natl. Acad. Sci. U. S. A.

[40] Dieci MV, Arnedos M, Andre F, Soria JC (2013). Fibroblast growth factor receptor inhibitors as a cancer treatment: from a biologic rationale to medical perspectives. Cancer Discov.

[41] Khan G, Moss R a, Braiteh F, Saltzman M (2014). Proactive strategies for regorafenib in metastatic colorectal cancer: implications for optimal patient management. Cancer Manag. Res.

[42] Mahadevan D, Cooke L, Riley C, Swart R, Simons B, Della Croce K, Wisner L, Iorio M, Shakalya K, Garewal H, Nagle R, Bearss D (2007). A novel tyrosine kinase switch is a mechanism of imatinib resistance in gastrointestinal stromal tumors. Oncogene.

[43] Gioia R, Leroy C, Drullion C, Lagarde V, Etienne G, Dulucq S, Lippert E, Roche S, Mahon F-X, Pasquet J-M (2011). Quantitative phosphoproteomics revealed interplay between Syk and Lyn in the resistance to nilotinib in chronic myeloid leukemia cells. Blood.

[44] Liu L, Greger J, Shi H, Liu Y, Greshock J, Annan R, Halsey W, Sathe GM, Martin A-M, Gilmer TM (2009). Novel mechanism of lapatinib resistance in HER2-positive breast tumor cells: activation of AXL. Cancer Res.

[45] Byers LA, Diao L, Wang J, Saintigny P, Girard L, Peyton M, Shen L, Fan Y, Giri U, Tumula PK, Nilsson MB, Gudikote J, Tran H (2013). An epithelial-mesenchymal transition gene signature predicts resistance to EGFR and PI3K inhibitors and identifies Axl as a therapeutic target for overcoming EGFR inhibitor resistance. Clin. Cancer Res.

[46] Heckmann D, Maier P, Laufs S, Li L, Sleeman JP, Trunk MJ, Leupold JH, Wenz F, Zeller WJ, Fruehauf S, Allgayer H (2014). The disparate twins: a comparative study of CXCR4 and CXCR7 in SDF-1α-induced gene expression, invasion and chemosensitivity of colon cancer. Clin. Cancer Res.

[47] Verma A, Warner SL, Vankayalapati H, Bearss DJ, Sharma S (2011). Targeting Axl and Mer kinases in cancer. Mol. Cancer Ther.

[48] Burbridge MF, Bossard CJ, Saunier C, Fejes I, Bruno A, Léonce S, Ferry G, Da Violante G, Bouzom F, Cattan V, Jacquet-Bescond A, Comoglio PM, Lockhart BP (2013). S49076 is a novel kinase inhibitor of MET, AXL, and FGFR with strong preclinical activity alone and in association with bevacizumab. Mol. Cancer Ther.

[49] Bardelli A, Parsons DW, Silliman N, Ptak J, Szabo S, Saha S, Markowitz S, Willson JK V, Parmigiani G, Kinzler KW, Vogelstein B, Velculescu VE (2003). Mutational analysis of the tyrosine kinome in colorectal cancers. Science.

[50] Boyd AW, Bartlett PF, Lackmann M (2014). Therapeutic targeting of EPH receptors and their ligands. Nat. Rev. Drug Discov.

[51] Montero JC, Seoane S, Ocaña A, Pandiella A (2011). Inhibition of SRC family kinases and receptor tyrosine kinases by dasatinib: possible combinations in solid tumors. Clin. Cancer Res.

[52] Brantley DM, Cheng N, Thompson EJ, Lin Q, Brekken R a, Thorpe PE, Muraoka RS, Cerretti DP, Pozzi A, Jackson D, Lin C, Chen J (2002). Soluble Eph A receptors inhibit tumor angiogenesis and progression in vivo. Oncogene.

[53] Dobrzanski P, Hunter K, Jones-Bolin S, Chang H, Robinson C, Pritchard S, Zhao H, Ruggeri B (2004). Antiangiogenic and antitumor efficacy of EphA2 receptor antagonist. Cancer Res.

[54] Carles-Kinch K, Kilpatrick KE, Stewart JC, Kinch MS (2002). Antibody targeting of the EphA2 tyrosine kinase inhibits malignant cell behavior. Cancer Res.

[55] Heemskerk B, Kvistborg P, Schumacher TNM (2013). The cancer antigenome. EMBO J.

[56] Gjertsen MK, Buanes T, Rosseland a R, Bakka a, Gladhaug I, Søreide O, Eriksen J a, Møller M, Baksaas I, Lothe R a, Saeterdal I, Gaudernack G (2001). Intradermal ras peptide vaccination with granulocyte-macrophage colony-stimulating factor as adjuvant: Clinical and immunological responses in patients with pancreatic adenocarcinoma. Int. J. Cancer.

[57] Overwijk WW, Wang E, Marincola FM, Rammensee H-G, Restifo NP (2013). Mining the mutanome: developing highly personalized Immunotherapies based on mutational analysis of tumors. J. Immunother. cancer.

[58] Brown SD, Warren RL, Gibb EA, Martin SD, Spinelli JJ, Nelson BH, Holt RA (2014). Neo-antigens predicted by tumor genome meta-analysis correlate with increased patient survival. Genome Res.

[59] Lawrence MS, Stojanov P, Polak P, Kryukov G V, Cibulskis K, Sivachenko A, Carter SL, Stewart C, Mermel CH, Roberts S a, Kiezun A, Hammerman PS, McKenna A (2013). Mutational heterogeneity in cancer and the search for new cancer-associated genes. Nature.

[60] Brahmer JR (2014). Immune checkpoint blockade: the hope for immunotherapy as a treatment of lung cancer?. Semin. Oncol.

[61] Naidoo J, Page DB, Wolchok JD (2014). Immune Checkpoint Blockade. Hematol. Oncol. Clin. North Am.

[62] Dolcetti R, Viel a, Doglioni C, Russo a, Guidoboni M, Capozzi E, Vecchiato N, Macrì E, Fornasarig M, Boiocchi M (1999). High prevalence of activated intraepithelial cytotoxic T lymphocytes and increased neoplastic cell apoptosis in colorectal carcinomas with microsatellite instability. Am. J. Pathol.

[63] Lipson EJ (2013). Re-orienting the immune system: Durable tumor regression and successful re-induction therapy using anti-PD1 antibodies. Oncoimmunology.

[64] Dienstmann R, Serpico D, Rodon J, Saura C, Macarulla T, Elez E, Alsina M, Capdevila J, Perez-Garcia J, Sánchez-Ollé G, Aura C, Prudkin L, Landolfi S (2012). Molecular profiling of patients with colorectal cancer and matched targeted therapy in phase I clinical trials. Mol. Cancer Ther.

[65] Manning G, Whyte DB, Martinez R, Hunter T, Sudarsanam S (2002). The protein kinase complement of the human genome. Science.

[66] Li H, Durbin R (2009). Fast and accurate short read alignment with Burrows-Wheeler transform. Bioinformatics.

[67] Li H, Handsaker B, Wysoker A, Fennell T, Ruan J, Homer N, Marth G, Abecasis G, Durbin R (2009). The Sequence Alignment/Map format and SAMtools. Bioinformatics.

[68] Kumar P, Henikoff S, Ng PC (2009). Predicting the effects of coding non-synonymous variants on protein function using the SIFT algorithm. Nat. Protoc.

[69] Adzhubei I, Jordan DM, Sunyaev SR (2013). Predicting functional effect of human missense mutations using PolyPhen-2. Curr. Protoc. Hum. Genet.

[70] Reva B, Antipin Y, Sander C (2011). Predicting the functional impact of protein mutations: application to cancer genomics. Nucleic Acids Res.

[71] Trapnell C, Pachter L, Salzberg SL (2009). TopHat: discovering splice junctions with RNA-Seq. Bioinformatics.

[72] Trapnell C, Williams BA, Pertea G, Mortazavi A, Kwan G, van Baren MJ, Salzberg SL, Wold BJ, Pachter L (2010). Transcript assembly and quantification by RNA-Seq reveals unannotated transcripts and isoform switching during cell differentiation. Nat. Biotechnol.

[73] Mortazavi A, Williams BA, McCue K, Schaeffer L, Wold B (2008). Mapping and quantifying mammalian transcriptomes by RNA-Seq. Nat. Methods.

[74] Marinov GK, Williams BA, McCue K, Schroth GP, Gertz J, Myers RM, Wold BJ (2014). From single-cell to cell-pool transcriptomes: stochasticity in gene expression and RNA splicing. Genome Res.

[75] Barretina J, Caponigro G, Stransky N, Venkatesan K, Margolin AA, Kim S, Wilson CJ, Lehár J, Kryukov G V, Sonkin D, Reddy A, Liu M, Murray L (2012). The Cancer Cell Line Encyclopedia enables predictive modelling of anticancer drug sensitivity. Nature.

[76] Segal NH, Parsons DW, Peggs KS, Velculescu V, Kinzler KW, Vogelstein B, Allison JP (2008). Epitope landscape in breast and colorectal cancer. Cancer Res.

[77] Reche PA, Reinherz EL (2007). Prediction of peptide-MHC binding using profiles. Methods Mol. Biol.

[78] Lundegaard C, Lamberth K, Harndahl M, Buus S, Lund O, Nielsen M (2008). NetMHC-3.0: accurate web accessible predictions of human, mouse and monkey MHC class I affinities for peptides of length 8-11. Nucleic Acids Res.

[79] Nielsen M, Lundegaard C, Lund O, Keşmir C (2005). The role of the proteasome in generating cytotoxic T-cell epitopes: insights obtained from improved predictions of proteasomal cleavage. Immunogenetics.

